# Diagnostic performance of different imaging modalities for splenic malignancies: A comparative meta-analysis

**DOI:** 10.1016/j.ejro.2024.100566

**Published:** 2024-04-22

**Authors:** Parya Valizadeh, Payam Jannatdoust, Mohammadreza Tahamtan, Hamed Ghorani, Soroush Soleimani Dorcheh, Khashayar Farnoud, Faeze Salahshour

**Affiliations:** a School of Medicine, Tehran University of Medical Science, Tehran, Iran; b Advanced Diagnostic and Interventional Radiology Research Center (ADIR), Tehran University of Medical Science, Tehran, Iran; c Department of Radiology, Shariati Hospital, Tehran University of Medical Sciences, Tehran, Iran; d Student's Scientific Research Center, Tehran University of Medical Science, Iran; e School of Medicine, Iran University of Medical Sciences, Tehran, Iran

**Keywords:** Splenic malignancy, PET, MRI, CT scan, Ultrasound

## Abstract

**Background and objectives:**

The spleen hosts both benign and malignant lesions. Despite multiple imaging modalities, the distinction between these lesions poses a diagnostic challenge, marked by varying diagnostic accuracy levels across methods. In this study, we aimed to evaluate and compare the diagnostic performance of various imaging techniques for detecting malignant splenic lesions.

**Methods:**

Following PRISMA guidelines, we searched PubMed, Scopus, and Web of Sciences databases for studies evaluating imaging techniques in detecting malignant splenic lesions. Data extraction included diagnostic accuracy metrics, and methodological quality was assessed using QUADAS-2. Diagnostic Test Accuracy meta-analyses were conducted using R (version: 4.2.1). Subgroup analyses and meta-regression were performed to compare different modalities and clinical settings.

**Results:**

Our study included 28 studies (pooled sample size: 2358), primarily using retrospective designs with histopathology as the reference standard. PET scan demonstrated the highest diagnostic accuracy (AUC: 92 %), demonstrating a sensitivity of 93 % (95 % CI: 80.4 % - 97.7 %) and a specificity of 82.8 % (95 % CI: 71.1 % - 90.4 %). Contrast-enhanced ultrasound (CEUS), Contrast-enhanced CT scan, and contrast-enhanced MRI also showed impressive performance with AUCs of 91.4 %, 90.9 %, and 85.3 %, respectively. Differences among these modalities were not statistically significant, but they outperformed non-contrast-enhanced methods. PET and CEUS exhibited higher specificity for lymphoma cases compared to studies including other malignancies.

**Conclusion and clinical implications:**

Overall, PET emerges as the best modality for splenic malignancies, and CEUS and CE-MRI show promise as potential alternatives, notably due to their reduced radiation exposure. Further research is essential for precise malignancy differentiation.

## Highlights

• PET emerges as the top diagnostic tool for splenic lesions.
• CE-US and CE-MRI offer comparable accuracy with reduced radiation.
• PET and CE modalities outperformed non-contrast methods.
• These findings highlight the potential of CE modalities as effective alternatives to PET.

## 1 Introduction

The spleen is the largest lymphoid organ of the human body. Although most splenic lesions are benign, the spleen might be involved in many malignancies, notably hematological malignancies. However, primary spleen malignancies are uncommon [1]. Since an extensive list of underlying diseases causes splenic lesions, it is necessary to provide proper radiologic diagnostic criteria to manage underlying pathologies appropriately. For instance, precise evaluation of splenic involvement in lymphoma significantly impacts tumor staging, therapeutic approach, and prognosis[2]. CT and ultrasonography are screening modalities among different modalities. MRI offers a sensitive technique for detecting lymphomatous involvement for staging Hodgkin’s disease and non-Hodgkin lymphomas. However, regarding sensitivity and specificity values, MRI should not be considered a replacement for conventional modalities but rather a complementary tool [3].

**Fig. 1:**
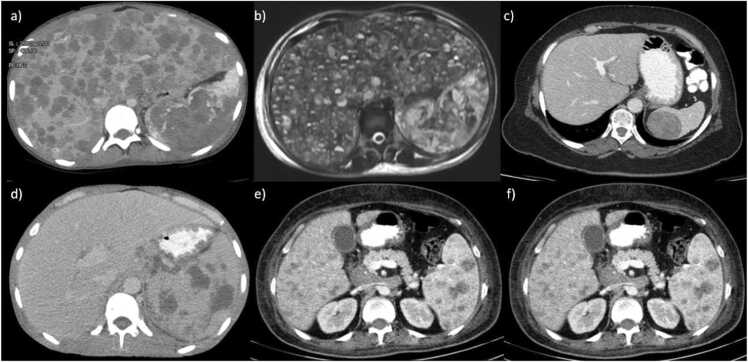
Examples of malignant splenic lesions identified through contrast-enhanced computed tomography (CT) and Magnetic Resonance Imaging (MRI). a) Axial contrast-enhanced CT scan of a 16-year-old female patient, revealing multiple hypodense lesions in the spleen and liver. Biopsy confirmed angiosarcoma. b) T2w MRI of a 21-year-old female patient, revealing multiple hypointense lesions in the spleen and liver. Biopsy confirmed angiosarcoma. c) Axial CT scan of a 56-year-old female patient demonstrating a 44 ×49 mm hypodense mass at the upper pole of the spleen. Subsequent Positron Emission Tomography (PET) scan uptake is suggestive of lymphoma, which was histopathologically confirmed. d) Axial CT scan of a 49-year-old male patient displaying multiple hypodense lesions within the spleen accompanied by splenomegaly, indicative of Hodgkin lymphoma, which was histopathologically confirmed. e) Portal phase axial CT scan of a 59-year-old female patient, revealing multiple hypodense lesions in the liver and spleen, indicative of lymphoma, which was clinically confirmed. f) Axial CT scan of a 58-year-old male patient showing a hypodense lesion with irregular borders in the spleen's posterior aspect, which was found in annual follow-up his colon cancer. Subsequent biopsy confirmed metastasis.

CT scans and MRIs are pivotal in distinguishing malignant splenic lesions from benign ones. Metastatic splenic lesions typically appear on CT as hypoattenuating masses, occasionally presenting cystic components, and exhibit low signal intensity on T1-weighted MRI sequences and high signal intensity on T2-weighted sequences, with contrast enhancement patterns varying based on the primary malignancy [4]. In the case of lymphoma, CT post-contrast imaging reveals that focal lesions are hypoenhancing relative to the splenic parenchyma, particularly noticeable in the late venous phase, and calcification within these lesions is rare but may occur post-treatment [2]. MRI features of lymphoma include well-defined masses that are low to iso-intense on T1 and T2 relative to the parenchyma, with focal lesions showing mild or no enhancement post-Gadolinium administration, indicating hypoenhancing lesions[4], [5], [6], and diffusion-weighted imaging (DWI) suggests restricted diffusion evidenced by relatively low ADC values [6]. Angiosarcomas are characterized on MRI by nodular hypointense masses on both T1- and T2-weighted images, with large masses showing increased signal intensity due to subacute hemorrhage or tumor necrosis, and areas of decreased signal intensity within tumors are attributed to chronic hemorrhage with hemosiderin deposition [7]. T1 gadolinium-enhanced imaging of angiosarcomas typically reveals intense and multinodular (heterogeneous) enhancement with focal areas of non-enhancement, likely indicative of intratumoral hemorrhage and necrosis [8]. Fig. 1 represents CT scans/MRIs of some patients from our institute with splenic lesions, which were later confirmed to be of malignant pathology through comprehensive clinical and histopathological data.

Contrast-enhanced ultrasonography (CEUS) is a novel modality, and some studies have reported it as a beneficial modality in discriminating splenic lesions[9], [10], [11], [12]. While CEUS is highly effective in detecting splenic malignancies and offers certain advantages over conventional ultrasonography (B-mode US), it presents a distinct challenge in interpretation due to the spleen’s unique characteristics. Unlike other organs, the spleen appears hyper-enhanced on CEUS, which can lead to malignant tissue exhibiting less enhancement than its surrounding normal tissue. This stands in contrast to most other organs, where malignant tissue usually shows more enhancement than adjacent healthy tissue, underscoring the intricacies of interpreting CEUS images of the spleen [12], [13].

Increased focal splenic uptake of FDG in FDG-PET/CT mainly suggests pathologic findings, including primary splenic neoplasm, metastasis, or inflammation/infection such as human immunodeficiency virus infection or infectious mononucleosis [14], [15]. On the other hand, the diffuse rise of splenic FDG uptake is usually regarded as an incidental finding, and its clinical importance remains imprecise [16]. Hybrid PET-CT imaging technique has emerged as the standard modality for initial staging, therapeutic response evaluation, and follow-up in patients with lymphoma [17]. One of the 18 F-FDG PET advantages over anatomic imaging such as CT and MRI is that it shows functional and metabolic changes and abnormalities that happen before anatomic abnormalities.

Overlapping imaging features challenge radiologists to distinguish between benign and malignant pathologies of the spleen lesions [18]. Some studies suggest that conventional imaging modalities cannot reliably differentiate benign from malignant splenic lesions, and no conclusions have been reached [18], [19], [20]. For instance, conventional ultrasonography and color Doppler sonography have restricted and insufficient diagnostic accuracy (30–75 %) in characterizing focal splenic lesions [13], [21], [22], [23].

Upon a thorough examination of the available literature, it becomes clear that there is a lack of consensus when determining the most effective diagnostic modality for assessing spleen involvement across different clinical scenarios. Therefore, the objective of this systematic review and meta-analysis is to comprehensively compare various imaging techniques, ultimately contributing to identifying the most appropriate modality for specific clinical contexts. We aim to assess the diagnostic performance of each imaging modality in detecting malignant splenic lesions, as well as comparing various modalities and identifying associated factors.

## 2 Methods

### 2.1 Search methods

Following the guidelines outlined in the PRISMA statement [24], a comprehensive literature search was carried out on April 28, 2023, utilizing the PubMed, Scopus, and Web of Science databases. Specific search terms were thoughtfully chosen for each database. Our initial database for the search was PubMed, and our systematic search strategy was as described below:

((splenic[title/abstract]) OR (spleen[title/abstract]) OR ("spleen"[MeSH Terms])) AND ((tumor*[title/abstract]) OR (cancer[title/abstract]) OR (neopla*[title/abstract]) OR (benign*[title/abstract]) OR (malignan*[title/abstract]) OR (“Neoplasms” [Mesh]) OR (“splenic neoplasms” [MeSH Terms]) OR (“Lymphoma” [Mesh])) AND ((“Positron-Emission Tomography” [Mesh]) OR (“Positron Emission Tomography Computed Tomography” [Mesh]) OR (“Magnetic Resonance Imaging” [Mesh]) OR (“Tomography, X-Ray Computed” [Mesh]) OR (“Ultrasonography” [Mesh]) OR (MRI) OR “CT scan” OR (“PET/CT”) OR (“PET CT”) OR (“PET-CT”) OR (ultrasound) OR “computed tomography” OR (sonography) OR “magnetic resonance”)

Following the PubMed search, we implemented a comparable search approach in both Scopus and Web of Science, making necessary adjustments to account for the technical variations in searching these databases. All findings were then exported to Endnote to eliminate duplicate records.

### 2.2 Study selection

The Rayyan tool [25] was utilized for paper screening. Unaware of each other’s findings, two independent researchers analyzed the titles and abstracts of every study to determine their suitability for inclusion. A third researcher then examined the outcomes. When disagreements arose, a collective decision about the study’s inclusion was made during a discussion. The studies deemed fit for inclusion were those original articles published after 1990 evaluating the ability of various imaging techniques and parameters to detect malignant spleen lesions or to identify splenic involvement in lymphoma patients. Review articles, case reports, unpublished studies, conference summaries, articles unavailable in English, instances where the full text couldn’t be accessed, and research involving animals were excluded from consideration.

### 2.3 Data extraction

Two investigators examined the full text of every selected article and gathered results related to

diagnostic test accuracy. The information obtained comprised a summary of study and patient characteristics, reference standard measure, sensitivity, and specificity, along with values for true positives (TP), false positives (FP), true negatives (TN), and false negatives (FN).

### 2.4 Assessment of methodological quality

To describe methodological quality in DTA systematic reviews, the QUADAS-2 tool is commonly used [26]. This tool encompasses 17 items across four domains: patient selection, the potential bias and relevance of the index test, the risk of bias and applicability of the reference standard test, and the flow and timing of the study. Each of the 17 items can be responded to with “yes,” “no,” or “unclear.”

### 2.5 Statistical analysis

The analysis involved extracting data from the included studies, providing sensitivity and specificity values. These values allowed us to calculate the numbers of TP, TN, FP, and FN for each imaging test. In cases where a study employed both contrast-enhanced (CE) and non-contrast-enhanced (NCE) versions of a particular test, data from both were extracted and utilized in distinct subgroups. Our analyses were stratified based on the type of imaging modality.

For studies that reported multiple interpretations of a single test, specifically involving different cut-off values, we included the interpretation with the highest reported accuracy, as indicated by the respective study. Subsequently, we created a comprehensive dataset, which served as the basis for conducting DTA meta-analyses for each modality individually.

To conduct these DTA meta-analyses, we followed the bivariate approach outlined by Reitsma et al. [27], which utilized logit-transformed sensitivity and false positive rate (FPR) data. We generated Summary Receiver Operating Curves (SROC) from these bivariate models to visually assess the performance of different modalities. The study-specific sensitivity and specificity point estimates in these SROC plots were scaled according to their respective weights in univariate diagnostic odds ratio random-effects models for each modality. Additionally, we reported the AUC and summary estimates for sensitivity and specificity based on the fitted bivariate models.

Regarding meta-regression and subgroup comparisons, we had predefined hypotheses. Specifically, we anticipated that factors such as the use of contrast enhancement and the choice of reference tests would significantly impact accuracy results. Consequently, we conducted subgroup analyses for these variables at the study level, both in univariate and multivariate models. We also compared the modalities of interest to each other.

Notably, some included studies focused on assessing the accuracy of imaging modalities for detecting splenic lymphoma in patients with known lymphoma, while others aimed to diagnose malignancy in focal splenic lesions. We hypothesized that these two groups would influence accuracy differently, so we included them as covariates in our analyses.

Currently, no established method exists for calculating the I2 statistic to assess heterogeneity in Diagnostic Test Accuracy (DTA) meta-analyses. However, we interpreted heterogeneity using the methods proposed by Holling et al. [28] and considered a subgroup to exhibit substantial heterogeneity if the upper bound of the I2 from Holling’s method exceeded 50 %.

We constructed double forest plots to visually represent study-specific sensitivity and specificity estimates. The confidence intervals in these forest plots were calculated using Wilson’s ranks score method [29]. Pooled effect sizes were derived from Reitsma’s model’s sensitivity, false positive rate (FPR), and their respective confidence intervals. The p-values for differences between subgroups, as visualized in the footnotes of the forest plots, were also derived from Reitsma’s bivariate model.

Finally, we employed funnel plots to assess publication bias, using logit-transformed sensitivity and FPR values for each modality. A quantitative asymmetry assessment was conducted using a generalization of Egger’s regression test method proposed by H. Noma [30] with 1000 bootstrap resamplings. All analyses were carried out using the R statistical programming language (version: 4.2.1) and the following packages: “mada”[31], “MVPBT”[32], “meta” [33], and “metafor” [34].

## 3 Results

### 3.1 Study selection

**Fig. 2:**
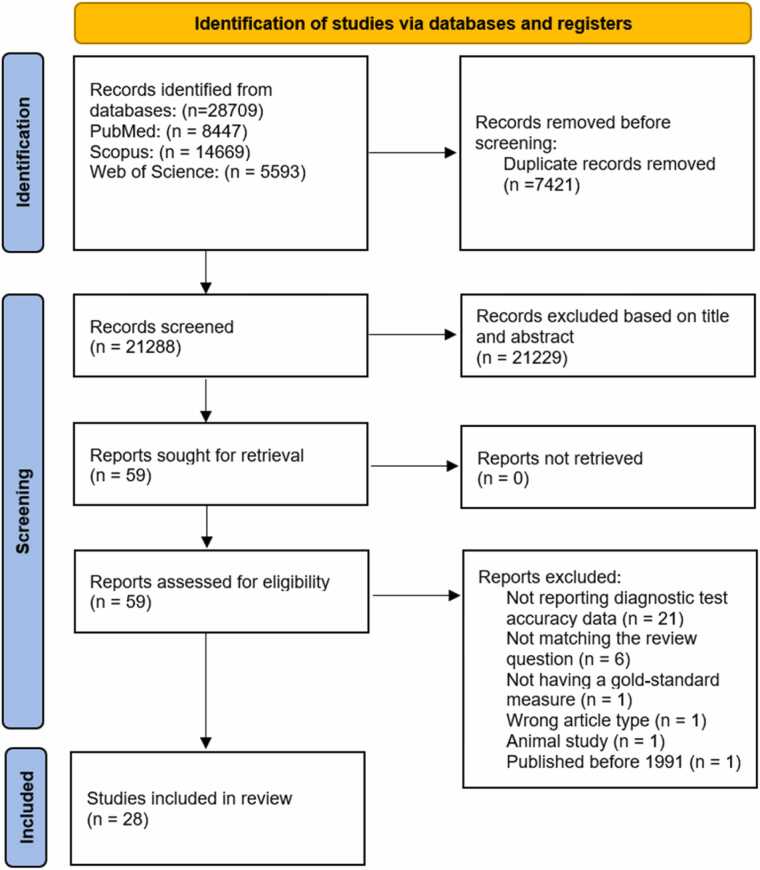
Study selection process depicted by PRISMA flowchart.

Employing a predefined search query, we retrieved 28,709 records from three databases (PubMed, Scopus, and Web of Science). After removing 7421 duplicates, 21,288 were screened based on titles and abstracts. Subsequently, 59 papers were identified as relevant and underwent full-text review. This led to the exclusion of 31 studies, resulting in the final inclusion of 28 studies in our analysis. The detailed selection process is available in Fig. 2**.**

### 3.2 Study characteristics

A total of 28 studies with a pooled sample size of 2358 were included in the study. The majority of the studies were retrospective in nature. Histopathological evaluation was the most common reference test, although several studies also employed a mix of histopathology, clinical evaluations, and imaging as their reference. The primary conditions assessed in the included studies were splenic lymphoma followed by focal splenic lesions.

Various imaging modalities and parameters were investigated as diagnostic criteria, including CE- and NCE-CT, plain US and CEUS, PET/CT, CE- and NCE-MRI in different sequences (like T1, T2, and DWI), and SPECT. Parameters such as splenic index thresholds, lesion size, and enhancement patterns were among the criteria used for diagnosis. Sensitivity and specificity values varied across studies and imaging modalities. These values differed based on the specific diagnostic criteria used in each study. More detailed information regarding gender, age, and other characteristics can be found in Table 1.

**Table 1:** Characteristics of the included studies.

Author, date	Country	Design	Sample size (M: B)	Male ( %)	Age	Reference test	Assessed condition	Modality and parameters under investigation	Sen, Spe ( %)
Aygun, 2004 [35]	USA	R	17 (8:9)	52.9	15.3, (10−20) (Mean, Range)	Histopathology	SL	NCE CT, SI> 500 + 20 × age (in years) cm3	50, 66
Von Herbay, 2009 [9]	Germany	P	35 (14:21)	68.5	54, 15 (Mean, SD)	mixed (histopathology and imaging)	FSL	1) CEUS, Early phase enhancement 2) CEUS, Enhancement at any stage 3) CEUS, Early enhancement followed by rapid washout	1) 71, 76 2) 71, 81 3) 91.4, 100
Hu, 2021 [59]	China	R	60 (40:20)	53.3	48.3, (15−79) (Mean, Range)	Histopathology	SL	1) PET/CT Tests in parallel (S/B >1.45 or S/L >2.42) 2) PET/CT, Tests in series(S/B >1.45 and S/L >2.42) 3) PET/CT, S/L>2.42 4) PET/CT, S/B>1.45	1) 82.5, 85 2) 57.5, 100 3) 62.5, 100 4) 77.5, 85
Jang, 2018 [38]	Korea	R	114 (66:48)	38.6	51.8, (12−79) (Mean, Range)	Histopathology	FSL	CE CT, lesion size> 4.25 cm	66.7, 72.7
Karunanithi, 2014 [42]	India	R	50 (26:24)*	58.8	41, 18.7, 46, (3−64) (Mean, SD, Median, Range)	Mixed (clinical and imaging)	Primary SL	1) PET/CT SUV max>2 2) PET/CT S/L>1.6 3) PET/CT, SA	1) 91.7, 73.7 2) 83.3, 100 3) 96.2, 91.7
Kharuzhyk, 2020 [60]	Belarus	P	92 (31:61)	51	44.7, 16.5 (Mean, SD)	Imaging (PET/CT)	SL	NCE-MRI (T1, T2, DWI), SA	54.8, 98.3
Lee, 2016 [48]	Korea	R	136 (11:125)	65	60.4, 15, (21−87) (Mean, SD, Range)	Mixed (histopathology and imaging)	SL	1) CT, ONHES 2) CT, Subjective splenomegaly 3) CT, Combined subjective splenomegaly and ONHES 4) CT, SI	1) 100, 91.2 2) 100, 84 3) 100,100 4) 95.9, 75.9
Littooij, 2015 [61]	Netherlands	P	107 (21:86)	64.5	42.7, 23.4, (6−72) (Mean, SD, Range)	Imaging (PET/CT)	SL	NCE MRI (T1, T2, DWI)	85.7, 96.5
Metser, 2005 [62]	Israel	R	88 (68:20)	57.9	(18−89) (Range)	Mixed (histopathology, imaging, and clinical)	FSL	Patients with known malignancy: 1)PET/CT, SA 2) PET/CT, SUV max>2.3 3) PET/CT, S/B>1.2 Patients without known malignancy: 4) PET/CT, SA 5) PET/CT, SUV max>2.2 6) PET/CT, S/B>1.5	1) 100, 100 2) 100,100 3) 100, 75 4) 100, 83 5) 100, 71 6) 100, 76
Picardi, 2022 [63]	Italy	R	260 (204:56)*	57	48, (22−72) (Median, Range)	Mixed (histopathology, imaging, and clinical)	SL	CEUS, SA	95, 100
Punwani, 2013 [53]	UK	R	31 (7:24)	45.1	15.9, (9.1–18.7) (Mean, Range)	Imaging (PET/CT)	SL	1) NCE MRI (T1, T2), SA 2) CE MRI (DCE, T1, T2), SA	1) 57, 100 2) 100, 100
Rini, 2002 [40]	USA	P	32 (12:20)	53.1	26 (Mean)	Mixed (clinical and imaging)	SL	1) PET, S/L>1 2) SPECT, S/L=1	1) 50, 95 2) 92, 100
Siniluoto, 1991[64]	Finland	R	61 (13:48)	N/A	N/A	Histopathology	SL	US, SA	54, 100
Stang, 2011 [11]	Germany	R	136 (78:58)*	58.8	51.5, (18−87) (Mean, Range)	Mixed (histopathology, imaging, and clinical)	FSL	1) CEUS mean 2) US mean	1) 92, 82.5 2) 75, 56
Tesero-tes, 1991[3]	Italy	P	74 (11:63)	48.6	35, (17−80) (Mean, Range)	Histopathology	SL	NCE MRI (T1, T2), SA	60, 97.7
Wan, 2000 [23]	Taiwan	R	53 (23:30)	69.8	50.8, (4−76) (Mean, Range)	mixed (histopathology, imaging, and clinical)	FSL	US, SA	100, 93.3
Yang, 2021 [12]	China	R	123 (40:83)	50.5	50.5, (19−83) (Mean, Range)	Mixed (histopathology and imaging)	FSL	1) US+CEUS (combining hypoechoic pattern, hypoenhancement pattern, and the presence of intralesional vessels) 2) US, SA 3) CEUS, SA	1) 55, 100 2) 61.3, 52.4 3) 93.8, 82.5
Yu, 2012 [13]	China	R	75 (19:56)*	58.3	47, 18.4, (18−82) (Mean, SD, Range)	Mixed (histopathology and imaging)	FSL	1) US, SA 2) CEUS, SA	1) 75, 84.2 2) 91.1, 95
Zytoon, 2020 [43]	Egypt	R	100 (69:31)	61	(20−70) (Range)	Histopathology	SL	1) CE CT, SA 2) PET/CT, SA	1) 68.1, 93.6 2) 100, 95.2
Munker, 1995 [54]	Germany	R	100 (36:64)	62	33.8 (Mean)	Histopathology	SL	1) US, SA 2) CECT, SA	1) 63, 98 2) 37, 96
Mainenti, 2012 [20]	Italy	R	54 (30:24)	51.9	54.8 (Mean)	Histopathology	FSL	1) CECT, Splenomegaly without focal lesions, and focal hypodense lesions 2) PET/CT, S/L>=1 or focal increased uptake	1) 100, 59 2) 100, 50
Berthelin, 2018 [49]	France	R	52 (26:26)	38.5	53.4, 12.2 (Mean, SD)	Histopathology	PC splenic involvement	1) CECT + CE-MRI, SA 2) CECT, SA 3) CE MRI (CE, T1, T2, DWI), SA	1) 92.3, 92.3 2) 84.6, 96.2 3) 84.6, 84.6
Dhyani, 2013 [65]	USA	R	53 (14:39)	49.1	19 (Mean)	Mixed (histopathology, imaging, and clinical)	FSL	PET/CT, SA	100, 67
De Jong, 2008 [66]	Netherlands	R	111 (32:79)	59.4	56.1, 16.7 (Mean, SD)	Mixed (histopathology and imaging)	SL	1) PET/CT, Combined S/L>1 and SI>2 SD 2) PET/CT, S/L>1 3) CT, SI, or spleen size>2 SD or presence of low-attenuation nodules	1) 100, 95 2) 75, 99 3) 91, 96
Choi, 2016 [67]	Korea	R	51 (16:35)	45.1	51.9, 13.0 (Mean, SD)	Mixed (histopathology and clinical)	FSL	1) MRI (CE, DWI), Combination of restricted diffusion and Low SI on 3-min delayed phase 2) CE-MRI, Low SI on 3-min delayed phase 3) NCE MRI (DWI), presence of diffusion restriction	1) 75, 100 2) 87.5, 85.7, 3) 81.3, 94.3
Abrishami, 2021[68]	Iran	R	161 (37:124)	54	59.7, 15.4 (Mean, SD)	Mixed (histopathology and imaging)	FSL	1) MRI (CE, T1, T2), lesion size>10.5 mm 2) NCE MRI (DWI), presence of diffusion restriction (ADC)	1) 76.1, 53.2 2) 50, 90.9
Cao, 2018 [69]	China	R	79 (20:59)	44.3	50 (Mean)	Histopathology	FSL	CT + MRI, Combining ill-defined borders and hypovascular enhancement pattern	75, 94.9
Jang, 2013 [70]	Korea	R	53	45.3	48.6 (Mean)	Mixed (histopathology and imaging)	FSL	1) MRI (CE, T1, T2, DWI) 2) MRI (CE, T1, T2), SA 3) CE MRI, Low SI on 3-min delayed phase	1) 90.9, 97.6 2) 18.2, 97.6 3) 90.9, 74

ADC: Apparent diffusion coefficient, B: Benign, CE: Contrast-enhanced, CEUS: Contrast-enhanced ultrasound, CT: Computed tomography, DCE: Dynamic Contrast-Enhanced, DWI: Diffusion-weighted imaging, FSL: Focal splenic lesion, M: Malignant, MRI: Magnetic Resonance Imaging, N/A: Not applicable, NCE: Non-contrast enhanced, P: Prospective, PC: Peritoneal carcinomatosis, PET: Positron Emission Tomography, R: Retrospective, S/B: Spleen/Bone marrow, S/L: Spleen/Liver, SA: Subjective assessment, SD: Standard deviation, Sen: Sensitivity, SI: Splenic index, SL: Splenic lymphoma, Spe: Specificity, SUV: Standardized uptake values, US: Ultrasound

* In these studies, the analysis was based on the number of splenic lesions.

### 3.3 Quality assessment

**Fig. 3:**
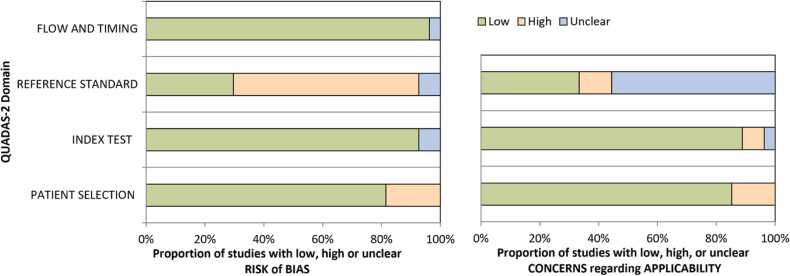
Summary results of critical risk of bias assessment using the QUADAS-2 tool.

The quality assessment outcomes utilizing the QUADAS-2 tool are illustrated in Fig. 3, while in-depth concerns for each of the seven domains assessed by QUADAS-2 for each study are detailed in Table 2. Predominantly, the risk of bias and applicability concerns were minimal in the majority of studies in the areas of patient selection, index test performance, and patient flow. However, numerous studies manifested significant concerns about the risk of bias in selecting and interpreting the reference test. This is attributed to using less reliable reference tests, such as clinical or imaging follow-ups, which may lead to an inflated perception of diagnostic accuracy since these reference tests could overlook some malignancies. Moreover, clarity was lacking in many studies about whether the conditions diagnosed by these references encompassed all malignancies. Histopathology was deemed the gold standard, and studies employing it exclusively as a reference exhibited low risks of bias and applicability concerns in the reference test domain. Given the prevalent issues in the reference test domain across various studies, a subgroup analysis was undertaken, when pertinent, to contrast studies with histopathology as the reference against those employing alternative reference tests to elucidate the potential effects of suboptimal reference tests.

**Table 2:** Study-specific findings of critical risk of bias assessment using the QUADAS-2 tool.

**Study**	**RISK OF BIAS**				**APPLICABILITY CONCERNS**		
	**PATIENT SELECTION**	**INDEX TEST**	**REFERENCE STANDARD**	**FLOW AND TIMING**	**PATIENT SELECTION**	**INDEX TEST**	**REFERENCE STANDARD**
Siniluoto, 1991							
Tesoro-Tess, 1991							
Munker, 1995							
Wan, 2000							
Rini, 2002							
Aygun, 2004							
Metser, 2005							
Von Herbay, 2009							
De Jong, 2009							
Stang, 2011							
Mainenti, 2012							
Punwani, 2013							
Dhyani, 2013							
Jang, 2013							
Karunanithi, 2014							
Littooij, 2015							
lee, 2016							
Choi, 2016							
Jang, 2018							
Cao, 2018							
Berthelin, 2019							
Kharuzhyk, 2020							
Zytoon, 2020							
Hu, 2021							
Yang, 2021							
Abrishami, 2021							
Picardi, 2022							

### 3.4 Meta-analysis results

**Fig. 4:**
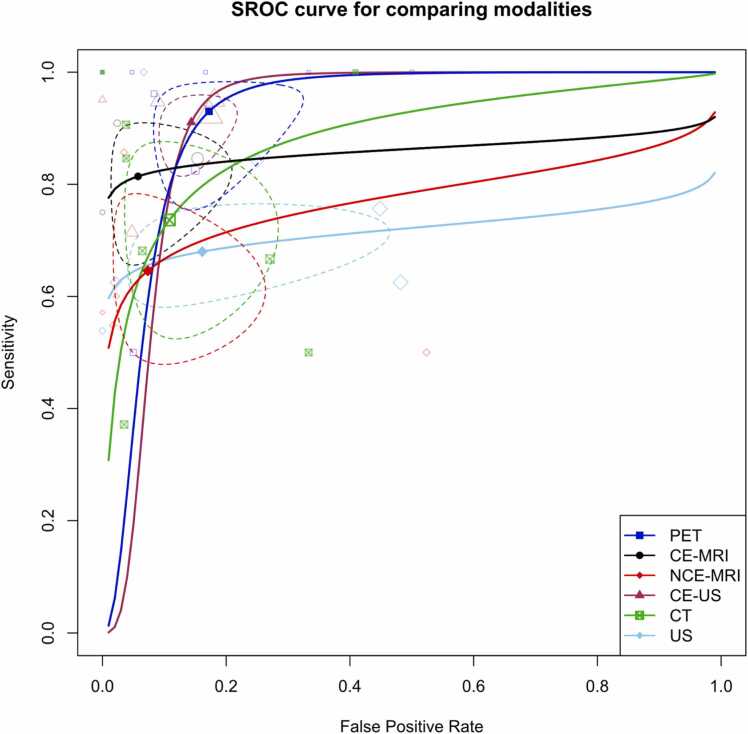
Summary receiver operating curve (SROC) plot for studies on primary included modalities, stratified by modality type, along with 95 % confidence regions and study-specific point estimates. CE: Contrast-enhanced. US: Ultrasound. SROC: Summary receiver operating curve.

Fig. 4 in our study illustrates the SROC curves for the six primary imaging modalities examined. In addition, it displays the pooled summary sensitivity and specificity values for each test, along with study-specific data points. To account for the expected substantial differences between CE- and NCE tests, we have separately visualized these tests for ultrasound and MRI modalities, where a sufficient number of included studies allowed for stratification based on these criteria.

Meta-regression was conducted to assess the impact of different modalities on the results. It was observed that the type of modality significantly explained the observed heterogeneity (p = 0.04). Furthermore, whether the study solely focused on splenic lymphoma was considered a relevant variable. As anticipated, the meta-regression for this variable demonstrated that it significantly contributed to the observed heterogeneity (p < 0.001). Specifically, notably higher specificity was exhibited by studies exclusively concentrating on splenic lymphoma (p < 0.001), regardless of the modality employed.

Another variable of interest included in our all-studies level meta-regression was the reference standard, which could be histopathology or other tests, including a combination of histopathology and other diagnostic methods. However, the meta-regression for the effect of this variable did not significantly explain the observed heterogeneity (p = 0.239).

Furthermore, in the bivariate model, when the effects of both modality and the study’s focus on lymphoma were assessed as covariates, both significant effects remained significant even after being controlled for by each other. The bivariate model provided a better explanation of the heterogeneity compared to each of the univariate models (p = 0.004 compared to the univariate lymphoma model and

#### 3.4.1 PET

**Fig. 5:**
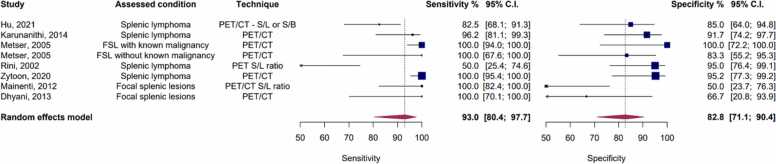
Paired forest plots of sensitivity and specificity of random effects bivariate-model meta-analysis of diagnostic accuracy of PET in detecting splenic malignancies. FSL: Focal Splenic Lesions. S/B: Spleen to bone marrow ratio. S/L: Spleen to liver ratio.

**Fig. 6:**
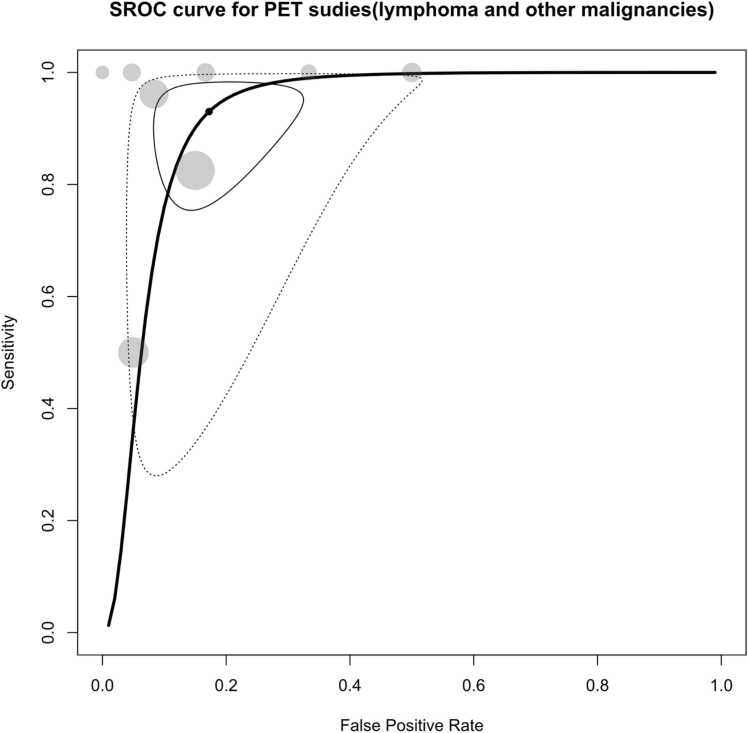
Summary receiver operating curve (SROC) plot for included PET studies, along with 95 % confidence regions, study-specific point estimates, and prediction region. SROC: Summary receiver operating curve.

In the random-effects bivariate-model meta-analysis comprising eight included studies evaluating the diagnostic accuracy of PET or PET/CT studies, the pooled sensitivity was determined to be 93 % (95 % CI: 80.4 % - 97.7 %), while the pooled specificity stood at 82.8 % (95 % CI: 71.1 % - 90.4 %). The AUC was calculated to be 92 %, as shown in Fig. 5, Fig. 6. Notably, substantial heterogeneity was observed among these studies, with Holling’s I2 ranging from 48.3 % to 69 %.

Upon conducting univariate meta-regression analyses, it was found that the histopathology reference standard did not significantly account for the observed heterogeneity. However, studies exclusively concentrating on lymphoma emerged as a significant explanatory factor for the observed heterogeneity (p = 0.036). Such studies demonstrated a notably higher specificity (p = 0.014).

For PET studies focused solely on the detection of splenic lymphoma, the pooled sensitivity, specificity, and AUC were determined to be 88 % (95 % CI: 60.1 % - 97.3 %), 89.3 % (95 % CI: 80.5 % - 94.6 %), and 90.4 %, respectively. On the other hand, in studies aimed at detecting malignancy in focal splenic lesions, the pooled sensitivity was 97.2 % (95 % CI: 89.5 % - 99.3 %), the pooled specificity was 69.8 % (95 % CI: 48.8 % - 84.9 %), and the pooled AUC reached 95.3 %. Supplementary figures 1 and 2 provide the corresponding forest and SROC plots for this subgroup analysis.

#### 3.4.2 CT

**Fig. 7:**
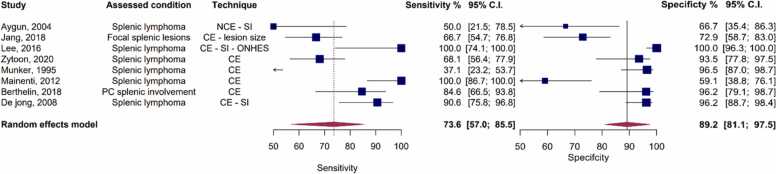
Paired forest plots of sensitivity and specificity of random effects bivariate-model meta-analysis of diagnostic accuracy of CT in detecting splenic malignancies. CE: Contrast-enhanced. ONHES: Obliteration of normal heterogeneous enhancement of the spleen. PC: Peritoneal carcinomatosis. SI: Splenic Index.

**Fig. 8:**
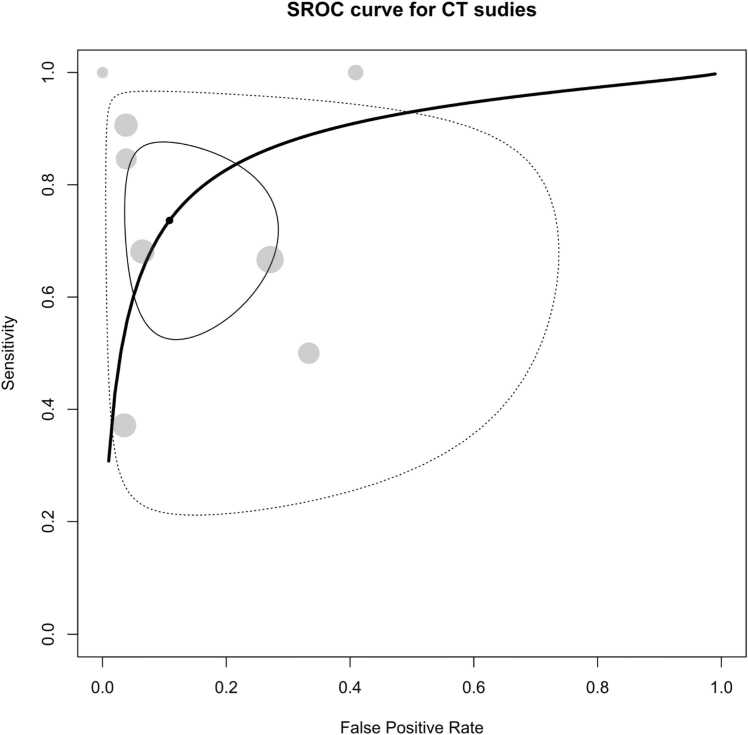
Summary receiver operating curve (SROC) plot for included CT studies, along with 95 % confidence regions, study-specific point estimates, and prediction region SROC: Summary receiver operating curve.

In the meta-analysis encompassing all CT studies, the pooled sensitivity was determined to be 73.6 % (95 % CI: 57 % - 85.5 %), accompanied by a pooled specificity of 89.2 % (95 % CI: 76.1 % - 95.5 %), and an AUC of 88 %, as depicted in Fig. 7, Fig. 8. It’s important to note that these studies exhibited high heterogeneity, with Holling’s I2 ranging from 50.2 % to 79.1 %.

Among the CT studies included in our analysis, the majority were CE studies, with only one under the NCE category [35]. After excluding this study, the sensitivity, specificity, and AUC for the CE-CT studies were 76.9 % (95 % CI: 58.8 % - 88.5 %), 90.9 % (95 % CI: 78.7 % - 96.4 %), 90.9 %, respectively. When considering the covariates in our assessment, the assessed condition of the studies (whether focusing on lymphoma or focal splenic lesions) did not emerge as a significant covariate (p = 0.12). However, the type of reference test was found to be a significant covariate for these studies (p = 0.012). Notably, when the reference test was other than histopathology, it predicted higher sensitivity (p = 0.046) and specificity (p = 0.023).

To provide further insights, we present the stratified forest and SROC plots for the CT studies based on the type of reference tests in supplementary figures 3 and 4. As illustrated, for studies utilizing histopathology as the reference test, the pooled sensitivity, specificity, and AUC were calculated as 67.5 % (95 % CI: 51.6 % - 80.2 %), 82.8 (95 % CI: 67.5 % - 91.8 %), and 81.2 %, respectively. In contrast, for the remaining studies, which encompassed those employing alternative reference tests or a combination of various reference tests, the pooled sensitivity was substantially higher at 90.6 % (95 % CI: 77.4 % - 96.4 %), along with a pooled specificity of 96.8 % (95 % CI: 91.7 % - 98.8 %), resulting in an impressive AUC of 96.5 %.

#### 3.4.3 CE and NCE MRI studies

**Fig. 9:**
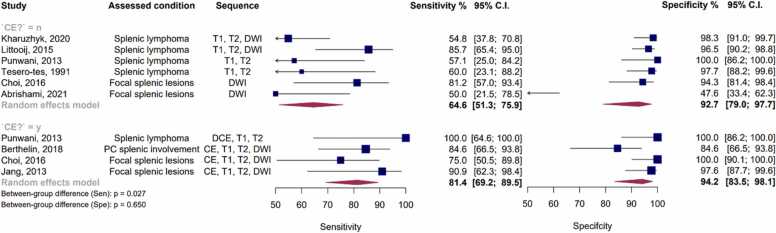
Paired forest plots of sensitivity and specificity of random effects bivariate-model meta-analysis of diagnostic accuracy of MRI in detecting splenic malignancies, stratified by contrast enhancement status. The between-subgroup difference statistics are derived from meta-regression using the bivariate Reitsma model. CE: Contrast-enhanced. PC: Peritoneal carcinomatosis. Sen: Sensitivity. Spe: Specificity.

**Fig. 10:**
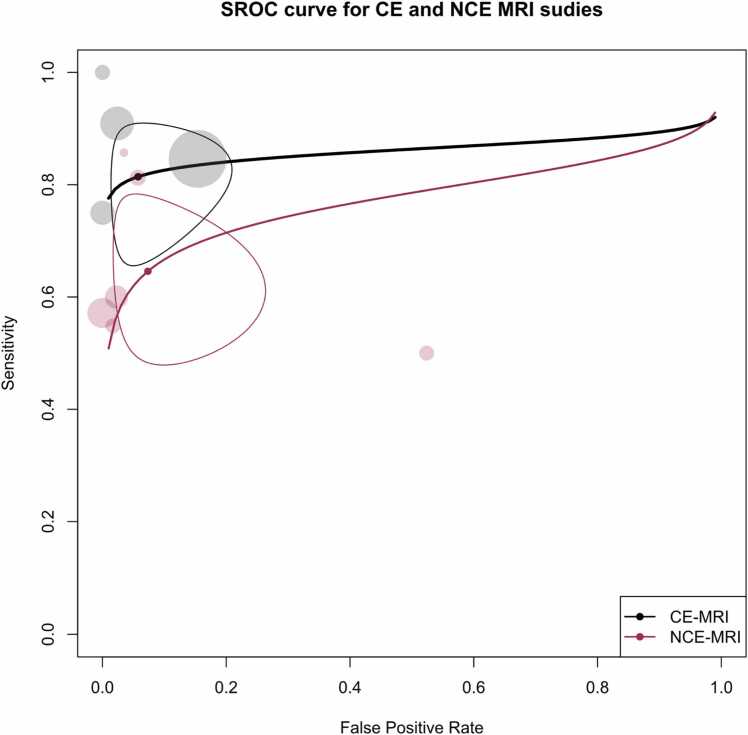
Summary receiver operating curve (SROC) plot for included MRI studies, stratified based on contrast enhancement status, along with 95 % confidence regions and study-specific point estimates. CE: Contrast-enhanced. SROC: Summary receiver operating curve.

Fig. 9, Fig. 10 present the forest plot and Summary Receiver Operating Characteristic (SROC) plot for both CE- and NCE-MRI studies. For CE-MRI sequences, the pooled sensitivity was calculated at 81.4 % (95 % CI: 69.2 % - 89.5 %), with a corresponding pooled specificity of 94.2 % (95 % CI: 83.5 % – 98.1 %) and an AUC of 85.3 %. In contrast, for NCE MRI studies, the pooled sensitivity, specificity, and AUC were determined to be 60.8 % (95 % CI: 47.3 % - 72.9 %), 92.8 % (95 % CI: 75.3 % - 98.2 %), and 70.3 %, respectively.

Significantly, a notable degree of heterogeneity was observed among all MRI studies, as indicated by Holling’s I2 values ranging from 51.8 % to 64.3 %. Notably, the presence of contrast enhancement was associated with higher sensitivity (p = 0.027) in these studies. Nevertheless, it is noteworthy that neither the choice of the reference test nor the exclusive focus on lymphoma emerged as significant covariates for MRI studies, both in the overall analysis and within each of the contrast enhancement subgroups.

#### 3.4.4 CE and NCE US studies

**Fig. 11:**
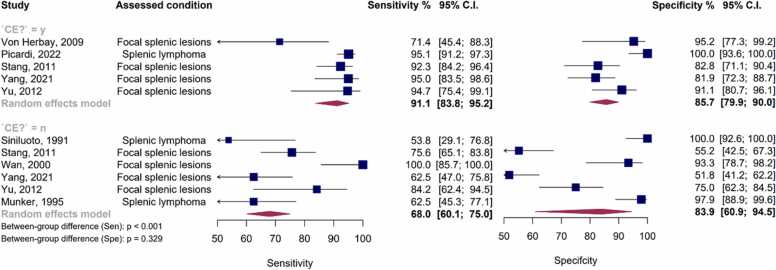
Paired forest plots of sensitivity and specificity of random effects bivariate-model meta-analysis of diagnostic accuracy of ultrasound in detecting splenic malignancies, stratified by contrast enhancement status. The between-subgroup difference statistics are derived from meta-regression using the bivariate Reitsma model. CE: Contrast-enhanced. Sen: Sensitivity. Spe: Specificity.

**Fig. 12:**
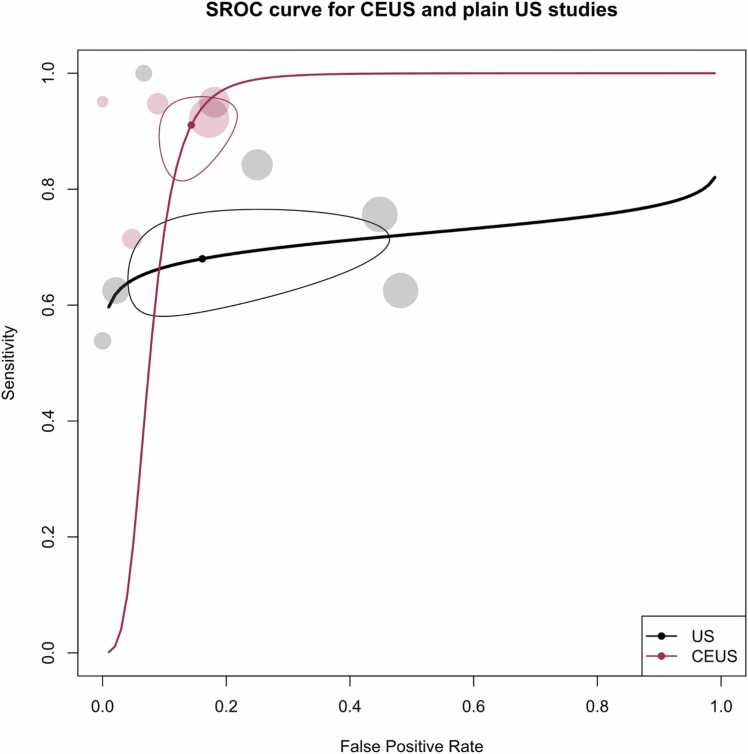
Summary receiver operating curve (SROC) plot for included ultrasound studies, stratified based on contrast enhancement status, along with 95 % confidence regions and study-specific point estimates. CE: Contrast-enhanced. SROC: Summary receiver operating curve. US: Ultrasound.

Fig. 11, Fig. 12 provide the forest and SROC plots for CEUS and NCE plain US studies. For CEUS sequences, the pooled sensitivity was estimated at 91.1 % (95 % CI: 83.8 % - 95.2 %), with a pooled specificity of 85.7 % (95 % CI: 79.9 % – 90.0 %) and an AUC of 91.4 %. In contrast, for NCE plain US studies, the pooled sensitivity, specificity, and AUC were found to be 68 % (95 % CI: 60.1 % - 75 %), 83.9 % (95 % CI: 60.9 % - 94.5 %), and 71.3 %, respectively.

Notably, a high level of heterogeneity was observed among all US studies, with Holling’s I2 values ranging from 79 % to 89.2 %. Moreover, the presence of contrast enhancement was significantly associated with higher sensitivity (p < 0.001).

Furthermore, both univariate and bivariate meta-regression models, controlled for the effect of contrast enhancement status, revealed the significance of the covariate “study being exclusively focused on lymphomas.” In greater detail, in the bivariate model controlled for CE status, this covariate was significantly associated with higher specificity (p < 0.001 in both models). The bivariate model demonstrated a superior ability to explain heterogeneity compared to univariate models (p < 0.001).

It’s worth mentioning that we refrained from conducting meta-regression due to the limited number of studies. Only two studies used histopathology as a reference, and both were categorized in the NCE-US group.

#### 3.4.5 The subgroup of contrast-enhanced studies

**Fig. 13:**
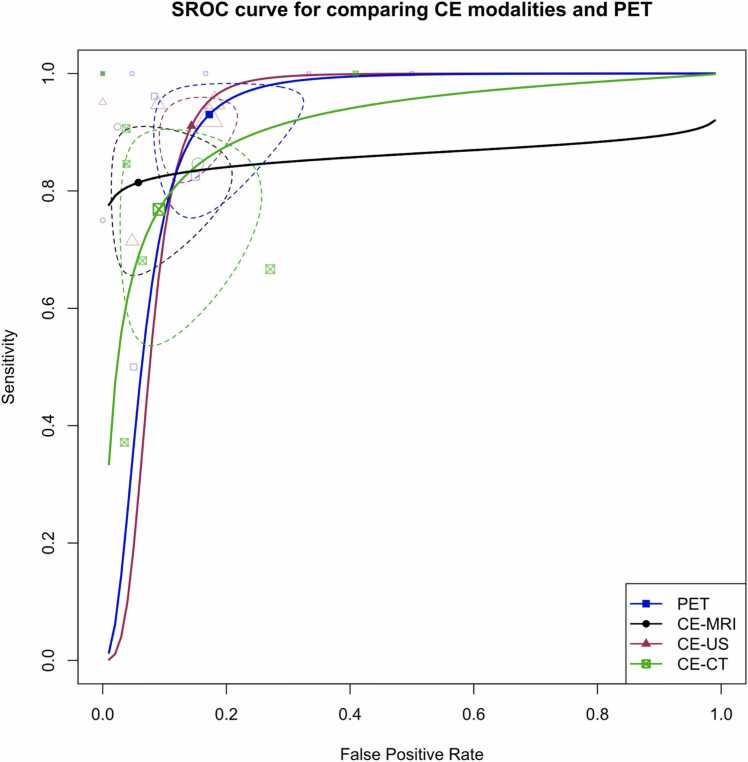
Summary receiver operating curve (SROC) plot for studies using contrast-enhanced (CE) modalities, stratified by modality type, along with 95 % confidence regions and study-specific point estimates. CE: Contrast-enhanced. US: Ultrasound. SROC: Summary receiver operating curve.

In light of observing the notable performance superiority of CE protocols over NCE tests, we conducted a focused analysis within this subset of studies. Fig. 13 illustrates the SROC plot, encompassing CE CT, MRI, US, and PET studies. The AUC values for these tests were calculated as 90.9 %, 85.3 %, 91.4 %, and 92 %, respectively.

Importantly, when conducting meta-regression to explore potential between-modality differences within this subset of studies, it was observed that there was no significant distinction between the modalities (p = 0.276).

#### 3.4.6 Publication bias

Supplementary figures 5–10 display the paired funnel plots of sensitivity and FPR rates for subgroups encompassing PET, CT, CE-MRI, NCE-MRI, CEUS, and US, respectively. Using the bivariate generalization of Egger’s test, our analysis revealed significant asymmetry in the CT, CEUS, and plain US subgroups. This suggests the potential presence of publication bias, with a significance level of P<0.001 for CT and P<0.05 for CEUS and US.

## 4 Discussion

In this systematic review and meta-analysis, we evaluated the diagnostic efficacy of various imaging modalities in distinguishing between malignant and benign splenic lesions while also conducting a comparative analysis of these modalities. The type of imaging modality significantly influenced diagnostic accuracy, with PET demonstrating a pooled sensitivity of 93 % and specificity of 82.8 %, CT showing a sensitivity of 73.6 % and specificity of 89.2 %, CE-MRI achieving a sensitivity of 81.4 % and specificity of 94.2 %, and CEUS exhibiting a sensitivity of 91.1 % and specificity of 85.7 %. Comparing AUC values, PET led with an impressive 92 %, followed by CEUS at 91.4 %, CT at 88 %, and CE-MRI at 85.3 %. Importantly, it should be noted that no significant differences were detected upon comparing CE sequences with PET, suggesting they are equally reliable. Studies exclusively focusing on splenic lymphoma consistently showed notably higher specificity. Furthermore, contrast enhancement notably improved sensitivity in CE-MRI and CEUS studies, emphasizing the impact of contrast enhancement. Additionally, the choice of reference standard impacted sensitivity and specificity in CT studies.

The discovery of spleen lesions often occurs incidentally, presenting a significant challenge for healthcare providers in determining the necessity of further diagnostic testing[36]. Early and precise differentiation between benign and malignant focal spleen lesions is crucial, as it informs timely and well-informed treatment decisions, guiding the choice between invasive and noninvasive interventions. While image-guided fine needle biopsy can provide a pathological diagnosis for various parenchymal organ lesions, thereby reducing the risk of unnecessary surgical morbidity, it carries a heightened risk of bleeding when inserted into highly vascularized tissues like the spleen [37]. Furthermore, the distinction between benign and malignant spleen tumors can be challenging due to overlapping imaging characteristics. Contextual factors such as the patient's clinical history, including recent trauma, body temperature, and immunological status, assume a pivotal role in characterizing incidental spleen lesions. Notably, features such as the solid structure of the mass, lymph node enlargement, and the potential presence of an underlying malignancy serve as valuable indicators for identifying malignant spleen lesions [38].

Recent advancements in molecular imaging, particularly 18 F-FDG PET/CT scans, have significantly improved the diagnosis, staging, and assessment of individuals with spleen lesions [39]. As previously highlighted, PET scans have demonstrated the highest diagnostic accuracy among the evaluated modalities for splenic malignancies, emphasizing the pivotal role of metabolic imaging. Importantly, metabolic abnormalities can arise from diffuse or focal tumor infiltration, even in the absence of noticeable morphological changes [40]. In cases where the diagnosis of malignant involvement in diffusely infiltrated organs like the spleen is required, typical indicators such as mass lesions or contrast enhancement may not be present. Consequently, PET/CT imaging has emerged as a superior alternative to CE-CT by enabling the visualization of metabolic activity [41]. Nevertheless, our subgroup meta-analysis and meta-regression in the current study revealed no significant differences among PET, CECT, CEUS, and CE MRI studies, shedding light on the comparability and potential non-inferiority of these imaging modalities in the context of spleen lesion assessment.

Notably, a significant difference emerged when comparing lymphoma patients to other groups, as studies exclusively focused on lymphoma demonstrated significantly higher specificity in their diagnostic assessments, while sensitivity remained comparable to that observed in studies involving other malignancies. This observation aligns with the generally heightened cellular metabolic activity associated with lymphoma, although certain low-grade lymphoma types can still yield false-negative results. Nevertheless, it is crucial to recognize that PET scans may introduce the potential for false positives and negatives due to the non-specific tracer, 18 F-FDG, which can accumulate in inflammatory and infectious conditions [42]. Notably, relying solely on size criteria for the morphological evaluation of lymph nodes in onco-hematology may prove inadequate, emphasizing the importance of integrating the metabolic status of lymph nodes through PET scans, even when their size appears normal on CT [43]. Furthermore, scenarios involving enlarged spleens without increased 18 F-FDG uptake can lead to false-negative results [44]. However, the high specificity exhibited by PET scans in the subgroup analysis strongly suggests that elevated FDG uptake in the spleen of patients with confirmed lymphoma highly indicates splenic lymphoma involvement, effectively distinguishing it from alternative pathological conditions.

Detecting morphological alterations, such as splenic enlargement or discrete tumor nodules, represents a fundamental requirement for CT to identify malignant involvement in the spleen. However, it's noteworthy that splenomegaly, often utilized as a diagnostic criterion for splenic lymphoma through CT scans, lacks specificity, as it can manifest due to various reactive processes unrelated to lymphoma, potentially leading to false-positive findings [45], [46]. On the other hand, focal hypodensities, although specific, are infrequently observed [40].

CT primarily serves the purpose of evaluating disease severity at the initial presentation and assessing disease recurrence post-treatment response [47]. While adopting arterial phase (AP) CT remains non-standard at certain institutions due to concerns regarding increased radiation exposure, it offers distinct advantages in accurately staging splenic involvement, particularly in lymphoma cases[48]. Nonetheless, given its wider availability and cost-effectiveness compared to MRI, CT appears to be a feasible initial imaging modality for splenic assessment [49]. Significantly, our subgroup meta-analysis revealed that studies employing histopathology as the reference test exhibited significantly lower sensitivity levels. This finding highlights the possibility that suboptimal reference tests utilized in various other studies could result in the potential underreporting of malignancies, thereby introducing a source of bias that may lead to the overestimation of sensitivity values.

In our current meta-analysis, CE-MRI emerged as the modality with the highest specificity among all imaging modalities. The diagnostic efficacy of MRI can be attributed to its ability to differentiate tissues based on factors such as water or fat content, perfusion characteristics, and water diffusibility [49]. Notably, CE-MRI demonstrated superior diagnostic efficacy compared to NCE-MRI in our study, aligning with prior research findings that underscore the enhanced accuracy of gadolinium-enhanced sequences in assessing splenic lymphoma[5]. Furthermore, some studies have highlighted the capacity of focal disease to manifest as hypointense lesions in contrast to the surrounding normal spleen tissue[5]. Moreover, diffusion-weighted MRI typically enhances the contrast between the hyperintense tumor and the adjacent normal tissue, aiding in the detection of peritoneal implants [50], [51], [52]. However, it's worth noting that the inherent hyperintense nature of the spleen on diffusion-weighted MRI has limited its potential for detecting perisplenic implants [49].

Given its comparable diagnostic performance to PET scans and other CE modalities, CE-MRI is a viable alternative when PET scans are inaccessible, particularly relevant in pediatric cases where the imperative to minimize ionizing radiation exposure is paramount. Whole-body MRI is a valuable option for staging lymphoma in children, although it may be time-consuming and not universally available [53]. Critical advantages of MRI in abdominal lymphoma staging include its multiplanar imaging capabilities and the associated minimal radiation exposure. However, it is essential to acknowledge cost considerations and potential challenges distinguishing bowel loops in some patients as inherent limitations[54]. It has been proposed that MRI should be considered for all incidentally discovered spleen lesions, as it can provide the radiologist with the tools needed to diagnose a range of benign conditions [55].

As revealed by the present study, CEUS demonstrated superior diagnostic efficacy compared to conventional US. The utilization of second-generation microbubble contrast agents has significantly advanced the capabilities of US in characterizing lesions by providing exceptional temporal and spatial resolution in various organs[56]. This technological enhancement, referred to as CEUS, plays a pivotal role in enhancing our ability to visualize the micro-perfusion of tissue and decipher vascularization patterns within lesions, effectively reducing background echoes originating from the spleen parenchyma [57].

Furthermore, our study revealed CEUS as a modality exhibiting comparable performance to other CE techniques. Nevertheless, a noteworthy advantage of CEUS over its counterparts lies in its suitability for patients with established renal or hepatic failure, allowing for the safe administration of microbubble contrast agents. Additionally, research has demonstrated that the second-generation microbubble contrast agent, SonoVue, induces spleen-specific enhancement that persists beyond the blood pool and liver enhancement phases. This extended enhancement window facilitates real-time monitoring of localized lesion characteristics, further enhancing the diagnostic utility of CEUS in clinical practice [57].

Similar to our findings within the PET scan subgroup, studies concentrating exclusively on lymphomas demonstrated significantly higher specificity across the US studies. Nevertheless, it is essential to acknowledge the existence of a learning curve associated with this relatively novel technique and the potential challenge posed by an overreliance on US operators. Moreover, ultrasound studies may suffer from variability in findings attributable to disparities among observers and equipment units. However, if CT or MRI imaging is not recommended, or when these techniques produce uncertain outcomes, CEUS emerges as a dependable and efficient substitute. It has the added advantages of ease of use, cost-effectiveness, and time-saving benefits, reinforcing its role in clinical practice[58].

## 5 Limitations

Our study is subject to several limitations. First, The diversity of splenic disorders included in our analysis, characterized by varying levels of diagnostic precision across different articles, posed a challenge to the homogeneity of our findings. In some instances, histologic confirmation was not obtained for all patients, introducing potential variability within the study groups. Second, differences in imaging protocols employed across various imaging centers and the inherent interobserver and interequipment variability added complexities to our analysis, potentially influencing the overall results. Third, specific subgroups lacked a sufficient number of published articles, limiting the generalizability of our subgroup analyses. These limitations should be considered when interpreting the findings of our meta-analysis and underscore the need for standardized protocols and additional research to address these sources of heterogeneity.

## 6 Conclusion

In our extensive systematic review and meta-analysis, we have delineated the diagnostic accuracies of various imaging techniques in differentiating benign from malignant splenic lesions. PET scans emerged as the modality with the highest diagnostic accuracy, showcasing the indispensable role of metabolic imaging in precisely characterizing spleen lesions. However, CE modalities such as CE-MRI also demonstrated commendable diagnostic efficacy, serving as viable alternatives in scenarios where PET scans are less accessible or not preferable. CEUS stood out for its comparable diagnostic performance to other CE techniques and its added advantage of significantly reduced radiation exposure. This characteristic of CEUS positions it as a particularly beneficial tool in pediatric cases and patients for whom radiation exposure is a critical concern without compromising the diagnostic accuracy required for effective clinical decision-making. Further studies are warranted to elucidate the differences among imaging modalities, especially their efficacy in distinguishing between various malignancies.

## Declaration of Generative AI and AI-assisted technologies in the writing process

During the preparation of this work, the authors used chatGPT in order to enhance the language and improve readability. After using this tool, the authors reviewed and edited the content as needed and took full responsibility for the content of the publication.

## Contribution of authors

All authors confirm their compliance with the current criteria for Authorship set forth by the International Committee of Medical Journal Editors (ICMJE).

## Funding

We declare that this study has not been supported by financial assistance or grants from any organizations.

## CRediT authorship contribution statement

**Parya Valizadeh:** Writing – review & editing, Writing – original draft, Supervision, Methodology, Data curation, Conceptualization. **Hamed Ghorani:** Writing – review & editing, Validation, Supervision. **Payam Jannatdoust:** Visualization, Methodology, Investigation, Formal analysis, Conceptualization. **Faeze Salahshour:** Supervision, Visualization, Writing – review & editing. **Khashayar Farnoud:** Writing – original draft, Investigation. **Soroush Soleimani Dorcheh:** Writing – original draft, Data curation. **Mohammadreza Tahamtan:** Writing – original draft, Data curation.

## Declaration of Competing Interest

The authors declare the absence of any known financial conflicts of interest or personal relationships that could have appeared to influence the work presented in this paper
