# Supplementary material for: Diagnostic performance of different imaging modalities for splenic malignancies: A comparative meta-analysis
Source: Eur J Radiol Open. 2024 Apr 22;12:100566. doi: 10.1016/j.ejro.2024.100566 (PMC11053287; doi:10.1016/j.ejro.2024.100566)
Supplement: Supplementary file 1 — Supplementary material [file mmc1.docx]

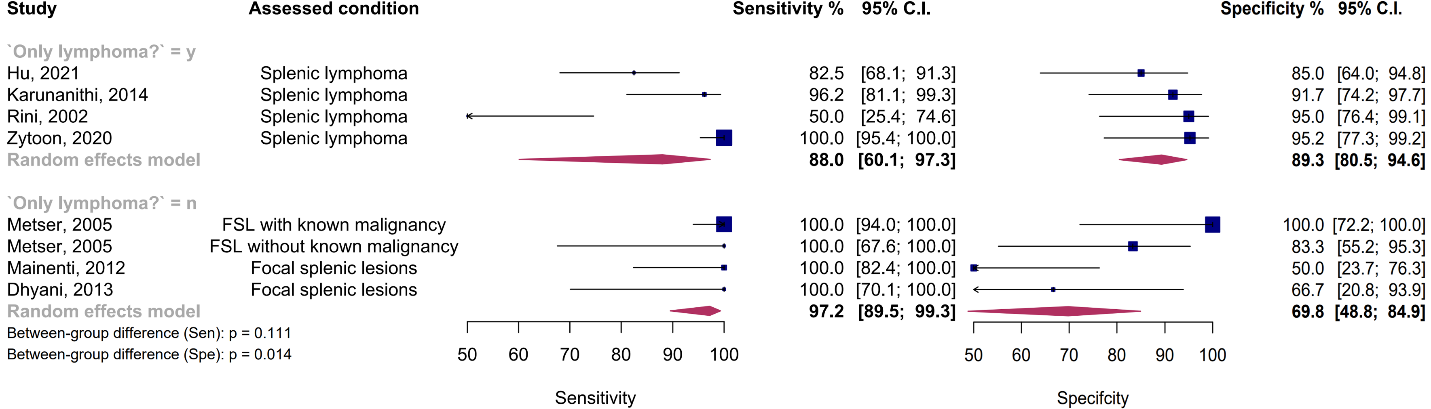


S. Figure 1. Paired forest plots of sensitivity and specificity of random effects bivariate-model meta-analysis of diagnostic accuracy of positron emission tomography (PET) in detecting splenic malignancies, stratified by the type of assessed condition. The between-subgroup difference statistics are derived from meta-regression using the bivariate Reitsma model. CI: Confidence interval. FSL: Focal splenic lesion.


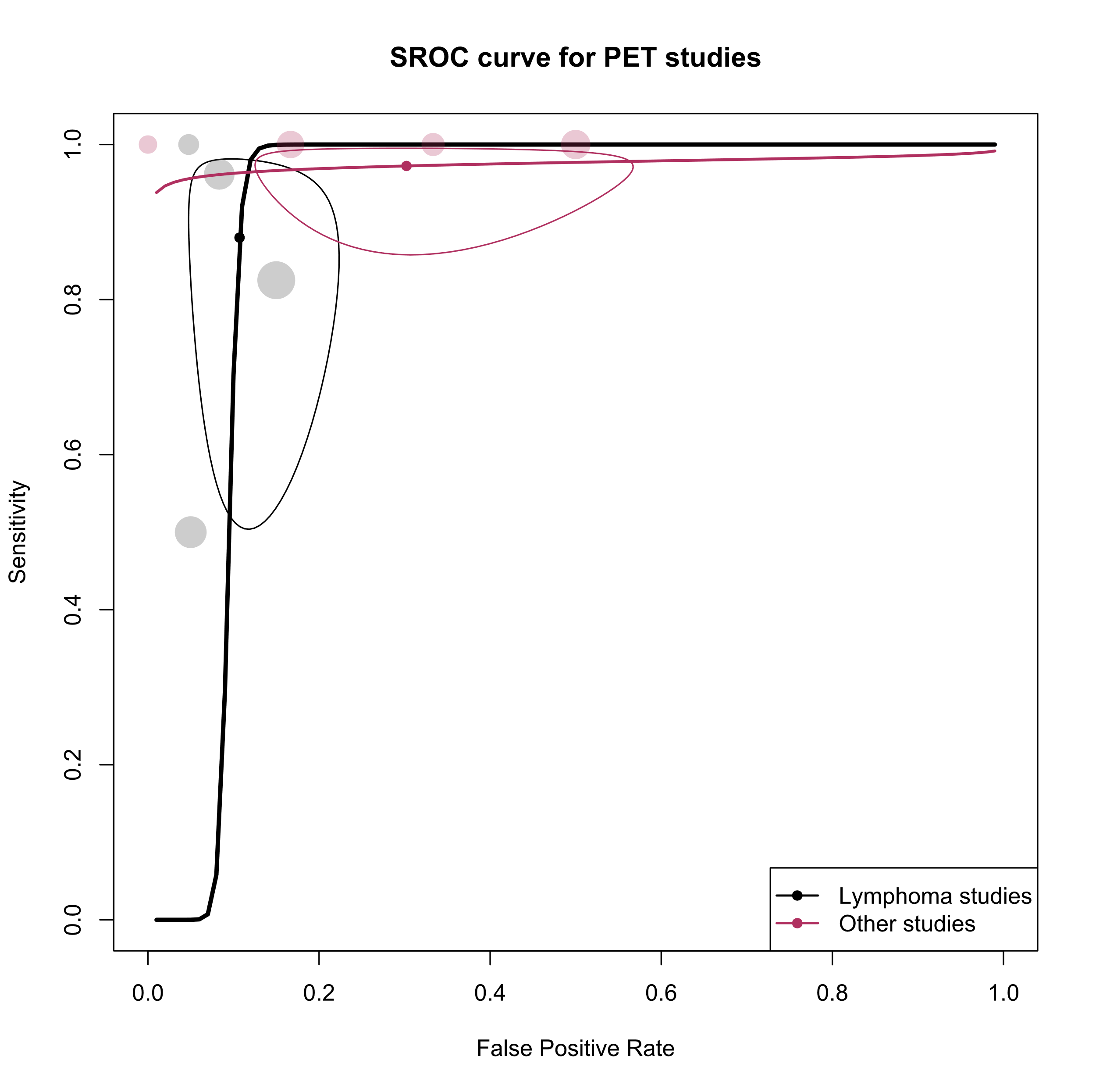


S Figure 2. Summary receiver operating curve (SROC) plots for included positron emission tomography (PET) studies, along with 95% confidence regions, study-specific point estimates stratified by the type of assessed condition. PET: Positron emission tomography. SROC: Summary receiver operating curve.


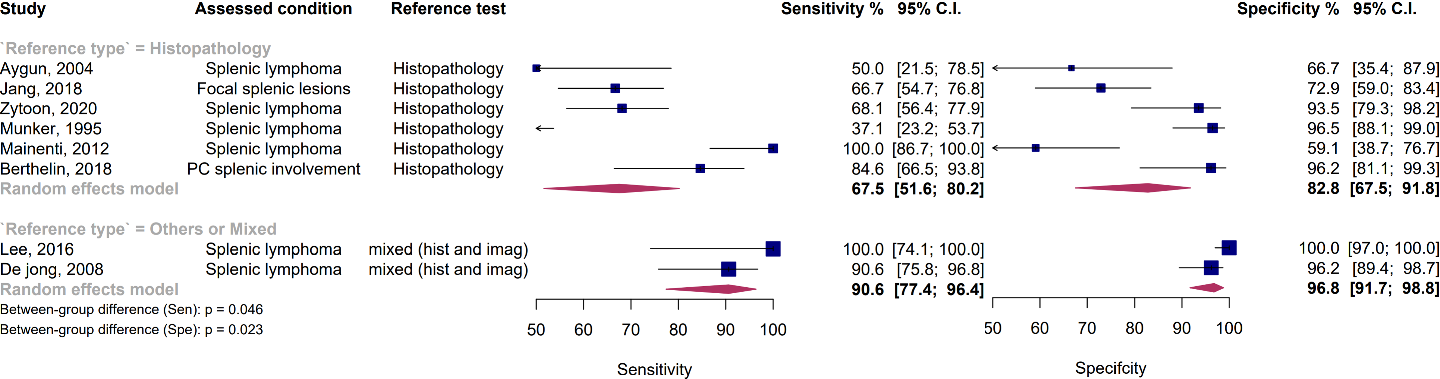


S. Figure 3. Paired forest plots of sensitivity and specificity of random effects bivariate-model meta-analysis of diagnostic accuracy of computed tomography (CT) in detecting splenic malignancies, stratified by type of reference tests. The between-subgroup difference statistics are derived from meta-regression using the bivariate Reitsma model. CI: Confidence interval. PC: Peritoneal carcinomatosis.


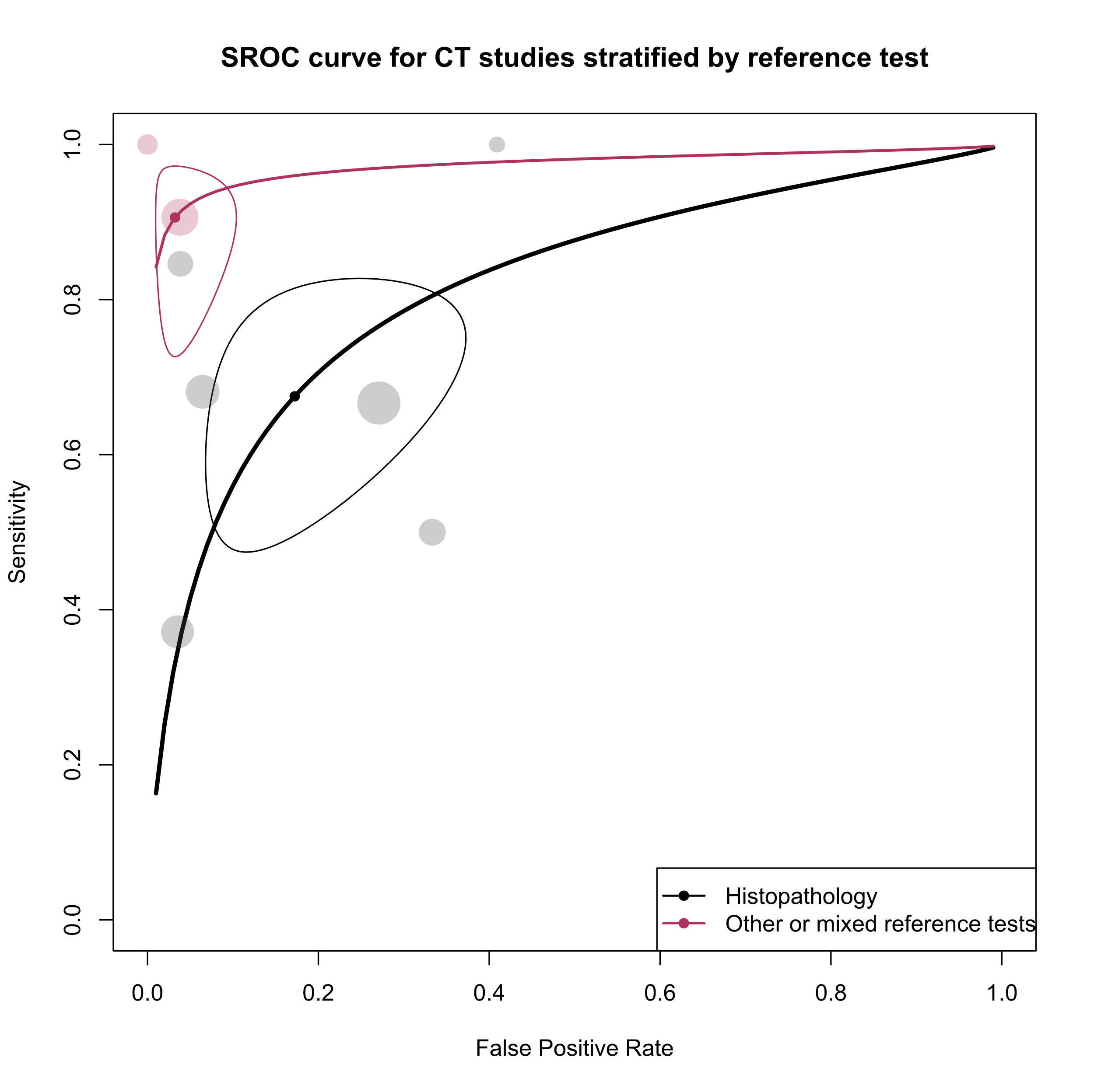


S Figure 4. Summary receiver operating curve (SROC) plots for included computed tomography (CT) studies and 95% confidence regions study-specific point estimates, stratified by categories of utilized reference tests. CT: Computed tomography. SROC: Summary receiver operating curve.


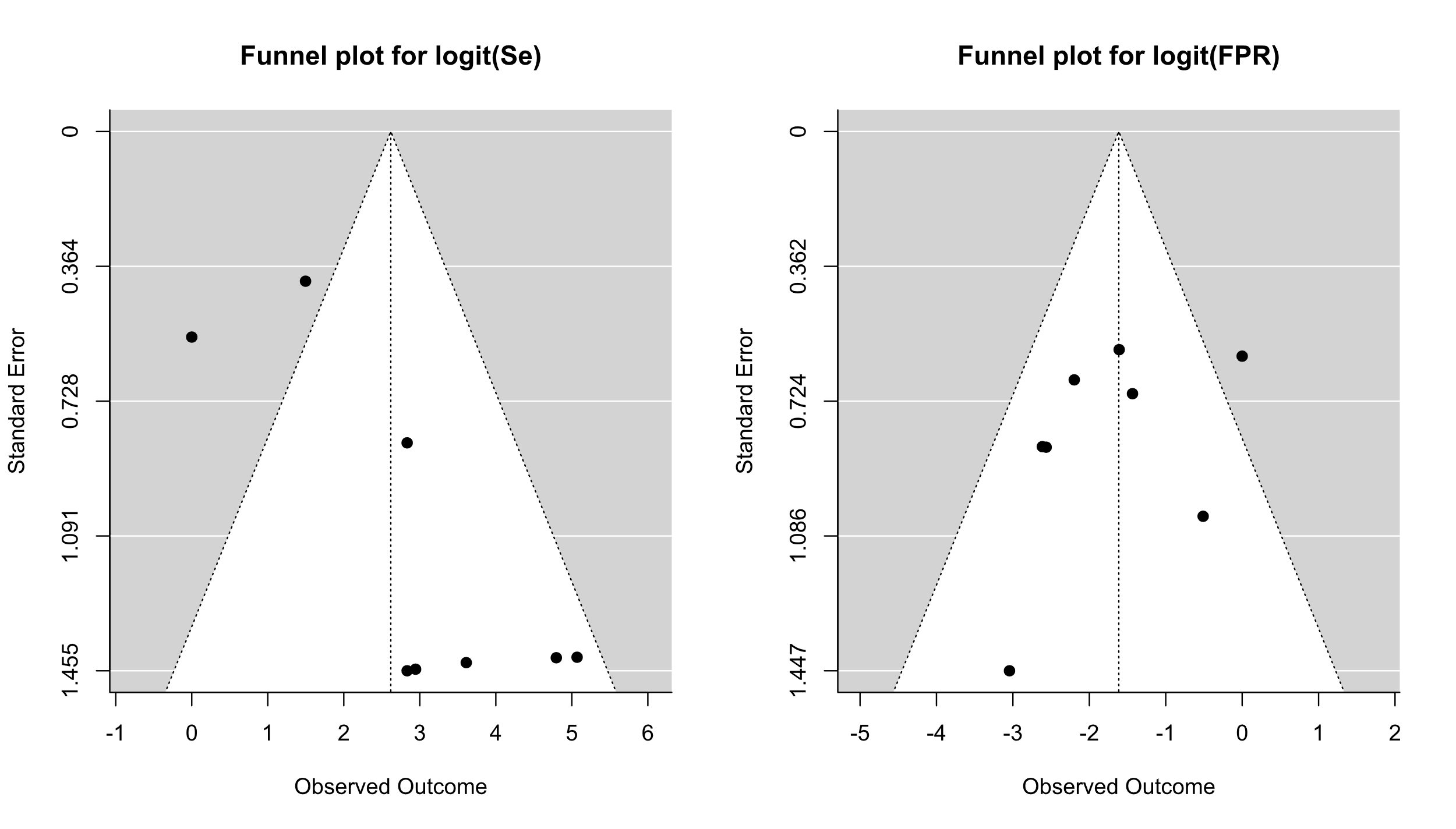


S Figure 5. Paired funnel plots for asymmetry assessment in reported sensitivity and specificity outcomes of included positron emission tomography (PET) studies. FPR: False positive rate. Se. Sensitivity.


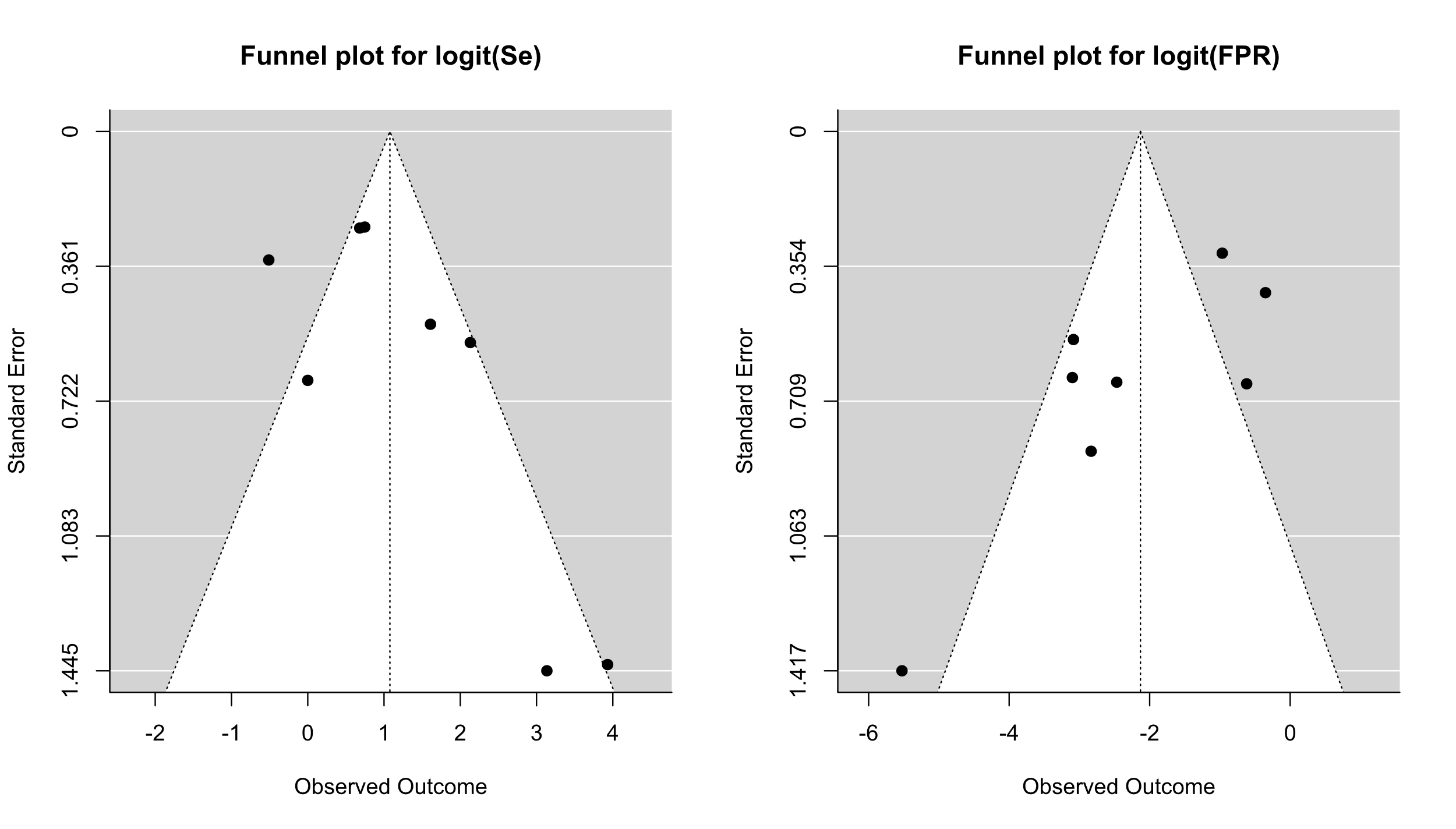


S Figure 6. Paired funnel plots for asymmetry assessment in reported sensitivity and specificity outcomes of included computed tomography (CT) studies. FPR: False positive rate. Se. Sensitivity.


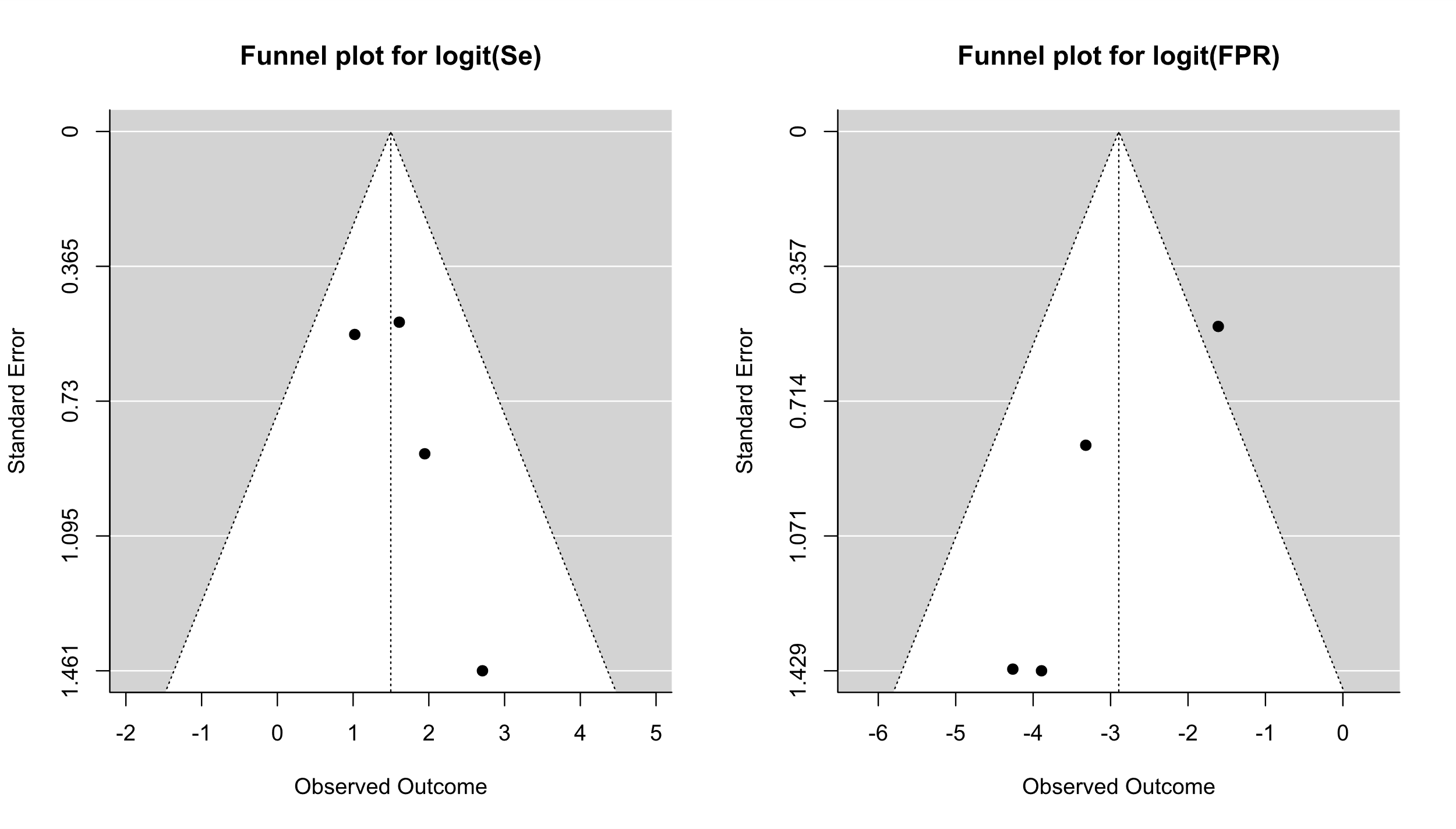


S Figure 7. Paired funnel plots to assess asymmetry in reported sensitivity and specificity outcomes of included contrast-enhanced magnetic resonance imaging (CE-MRI) studies. FPR: False positive rate. Se. Sensitivity.


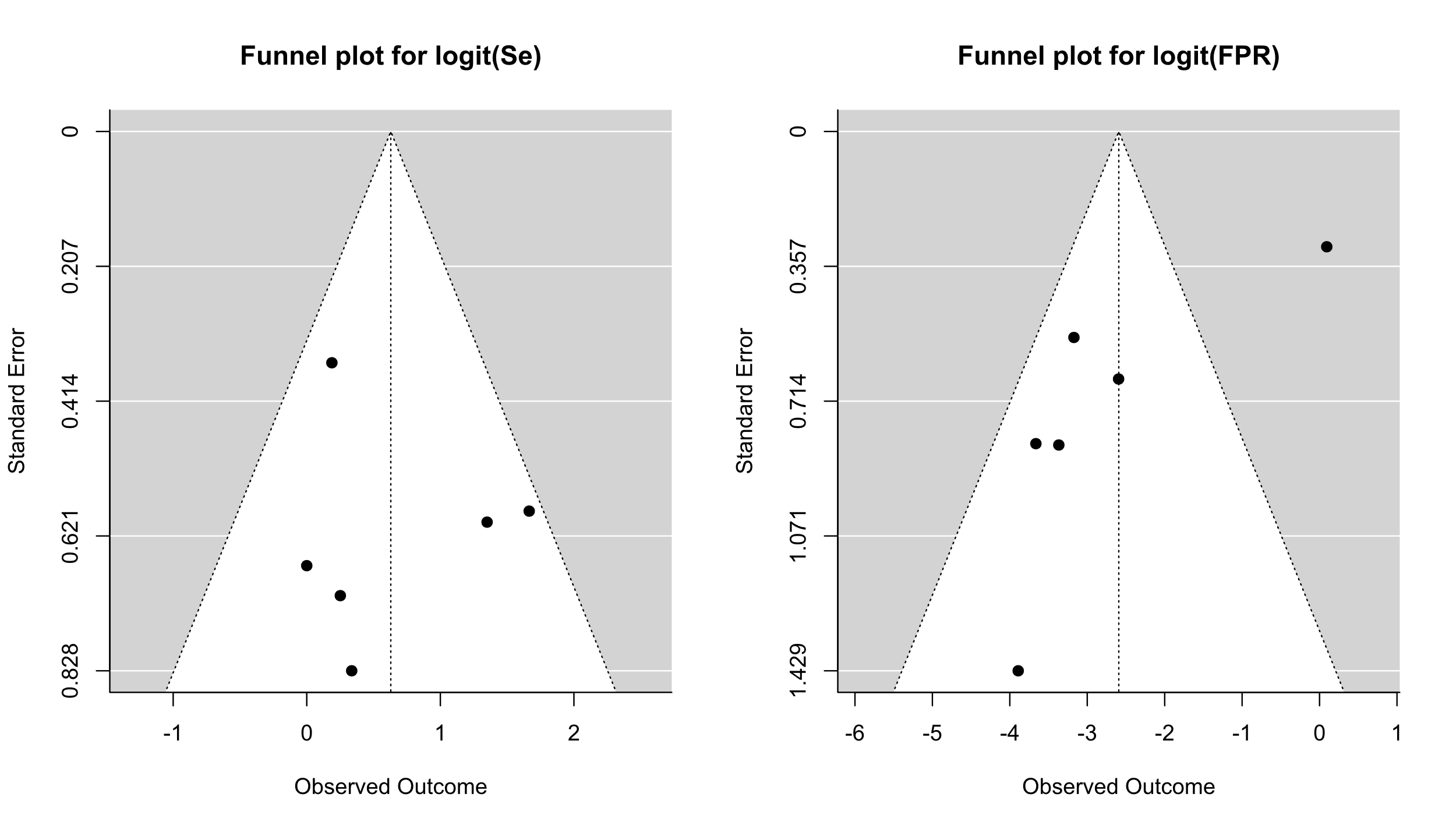


S Figure 8. Paired funnel plots to assess asymmetry in reported sensitivity and specificity outcomes of included non-contrast-enhanced magnetic resonance imaging (NCE-MRI) studies. FPR: False positive rate. Se. Sensitivity.


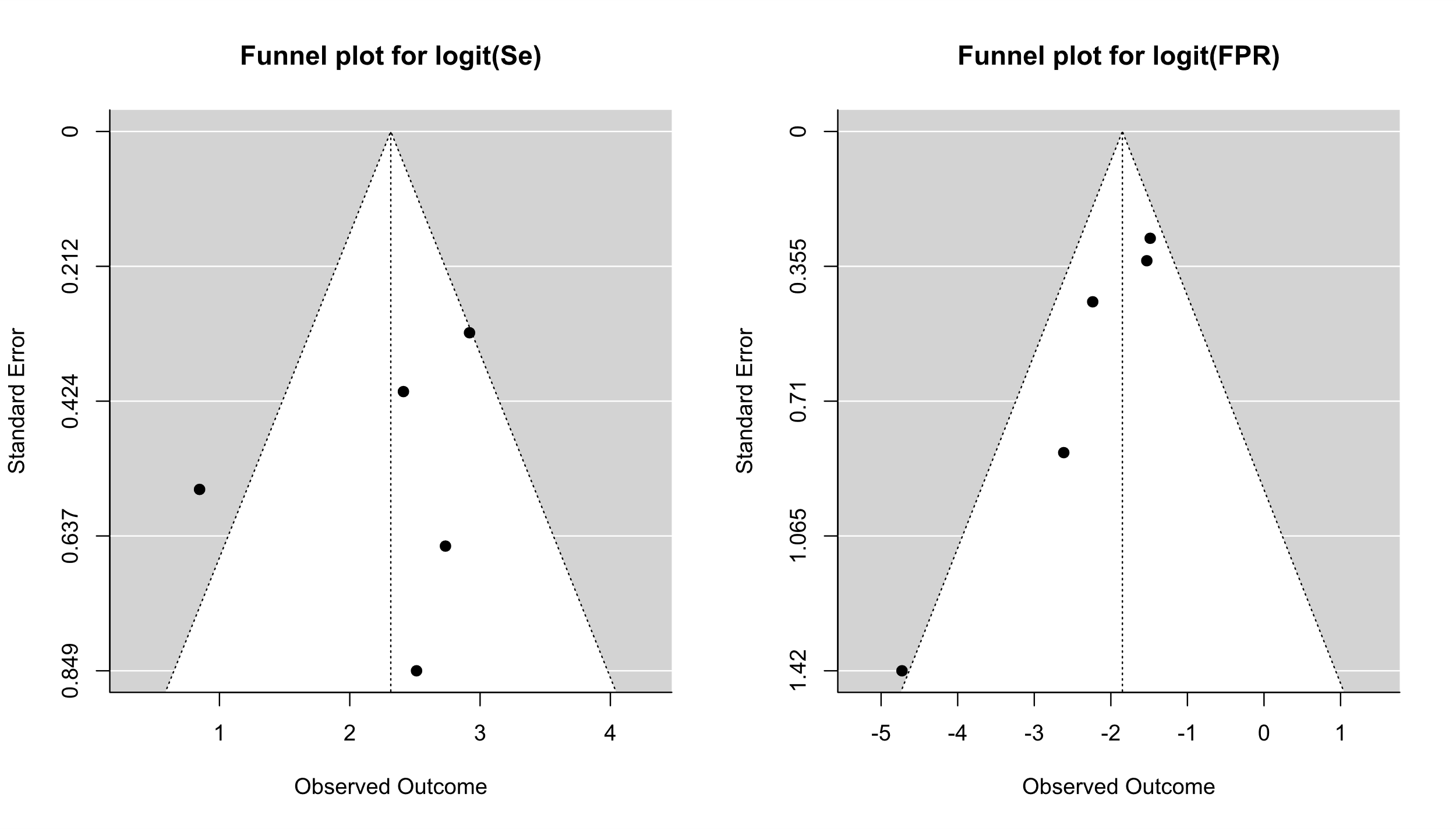


S Figure 9. Paired funnel plots to assess asymmetry in reported sensitivity and specificity outcomes of included contrast-enhanced ultrasound (CE-US) studies. FPR: False positive rate. Se. Sensitivity.


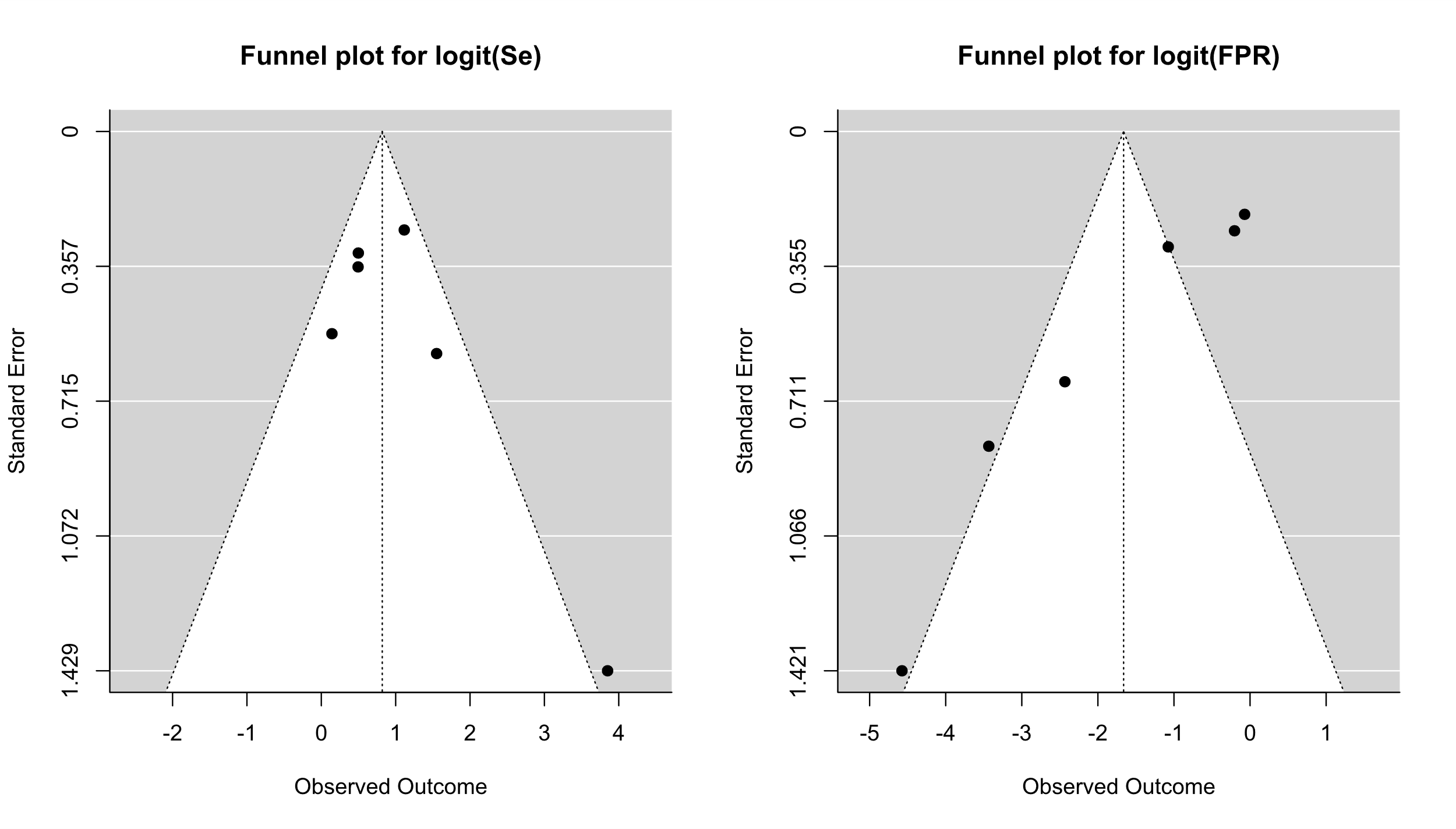


S Figure 10. Paired funnel plots to assess asymmetry in reported sensitivity and specificity outcomes of included non-contrast-enhanced ultrasound (NCE-US) studies. FPR: False positive rate. Se. Sensitivity.
